# Long-term low-dose sirolimus therapy and successful discontinuation in an adult with kaposiform lymphangiomatosis and disseminated intravascular coagulation: a case report

**DOI:** 10.3389/fmed.2026.1852990

**Published:** 2026-05-25

**Authors:** Junya Liu, Whenshuai Xu, Qingwei Jiang, Kai-Feng Xu

**Affiliations:** 1 Department of Pulmonary and Critical Care Medicine, Peking Union Medical College Hospital, Chinese Academy of Medical Sciences and Peking Union Medical College, Beijing, China; 2 Department of Pulmonary and Critical Care Medicine, Beijing Tsinghua Changgung Hospital, Beijing, China; 3 Department of Gastroenterology, Peking Union Medical College Hospital, Chinese Academy of Medical Sciences and Peking Union Medical Science, Beijing, China; 4 State Key Laboratory of Complex Severe and Rare Diseases, Peking Union Medical College Hospital, Chinese Academy of Medical Sciences and Peking Union Medical College, Beijing, China

**Keywords:** disseminated intravascular coagulation, kaposiform lymphangiomatosis, mTOR inhibitor, *NRAS* p.Q61R mutation, sirolimus

## Abstract

Kaposiform lymphangiomatosis is a rare, aggressive lymphatic anomaly frequently complicated by disseminated intravascular coagulation. We report a 23-year-old male with a confirmed *NRAS* p.Q61R mutation who was treated with low-dose sirolimus (1 mg/day, trough 4–5 ng/mL). Significant clinical, laboratory, and radiologic improvements were observed over four years of therapy. Following discontinuation, the patient remained stable throughout five years of follow-up. This case suggests that long-term low-dose sirolimus can effectively control disease progression, and that carefully monitored treatment withdrawal may be feasible.

## Introduction

Kaposiform lymphangiomatosis (KLA) is a rare, rapidly progressive lymphatic anomaly with high morbidity and mortality, frequently associated with disseminated intravascular coagulation (DIC) (1, 2). Initially classified as a subtype of generalized lymphatic anomaly (GLA) by the International Society for the Study of Vascular Anomalies (ISSVA) (3, 4), KLA is now recognized as a distinct entity (5).

The disease predominantly affects children but can also present in adolescents and adults (1, 2, 6). Histologically, KLA features infiltrative proliferation of spindle-shaped lymphatic endothelial cells. Commonly involved sites include the mediastinum, lungs, bones, and retroperitoneum (2). Clinical manifestations depend on site involvement and often include respiratory symptoms, effusions, splenomegaly, and coagulopathy (2, 7, 8).

KLA is strongly associated with somatic *NRAS* p.Q61R mutations, which aberrantly activate both the PI3K/AKT/mTOR and RAS/MAPK signaling pathways, promoting abnormal lymphatic proliferation and malformed lymphatic networks (6, 9–12). Conventional treatments, including vincristine, corticosteroids, thalidomide, and surgical interventions, show limited efficacy (7).

The mTOR inhibitor sirolimus has demonstrated therapeutic potential in complex lymphatic anomalies, including KLA (5, 13, 14). However, optimal dosing, monitoring strategies, long-term efficacy, and protocols for treatment discontinuation remain unclear, particularly in adult patients (2, 8, 9, 15).

Here, we report an adult patient with KLA and DIC successfully treated with long-term low-dose sirolimus, achieving durable remission after drug withdrawal. This case highlights individualized dosing, therapy duration, and discontinuation strategies for KLA.

## Case presentation

A 23-year-old previously healthy male presented with a history of recurrent diarrhea since 2013, occurring 3–4 times per day, characterized by yellow, pasty stools without blood, mucus, or tenesmus, accompanied by abdominal bloating. In 2015, colonoscopy suggested nonspecific colitis, and a one-month course of mesalazine produced no improvement. By 2016, he had lost approximately 10 kg and developed recurrent cutaneous ecchymoses and abdominal distension.

Extensive investigations, including upper and lower gastrointestinal endoscopy, contrast-enhanced CT, PET-CT, and multiple biopsies (inguinal, retroperitoneal, mesenteric, and sigmoid mesentery), revealed soft tissue thickening in the mediastinum and retroperitoneum, and PET-CT showed mild metabolic activity (SUVmax 1.4–2.6). Colonoscopy demonstrated diffuse edema, bleeding, and mucosal friability from the sigmoid colon to the rectum, while gastroscopy revealed submucosal ecchymoses in the stomach and duodenum.

Laboratory studies revealed progressive thrombocytopenia (nadir 26 × 10^9^/L), prolonged prothrombin time (17.0 s) and APTT (47.6 s), low fibrinogen (0.61 g/L), and elevated D-dimer (15.38 mg/L). The DIC score was ≥7, meeting diagnostic criteria. Comprehensive immunologic, infectious, hematologic, and tumor evaluations were unremarkable. A systemic lymphatic anomaly was suspected.

**Figure 1:**
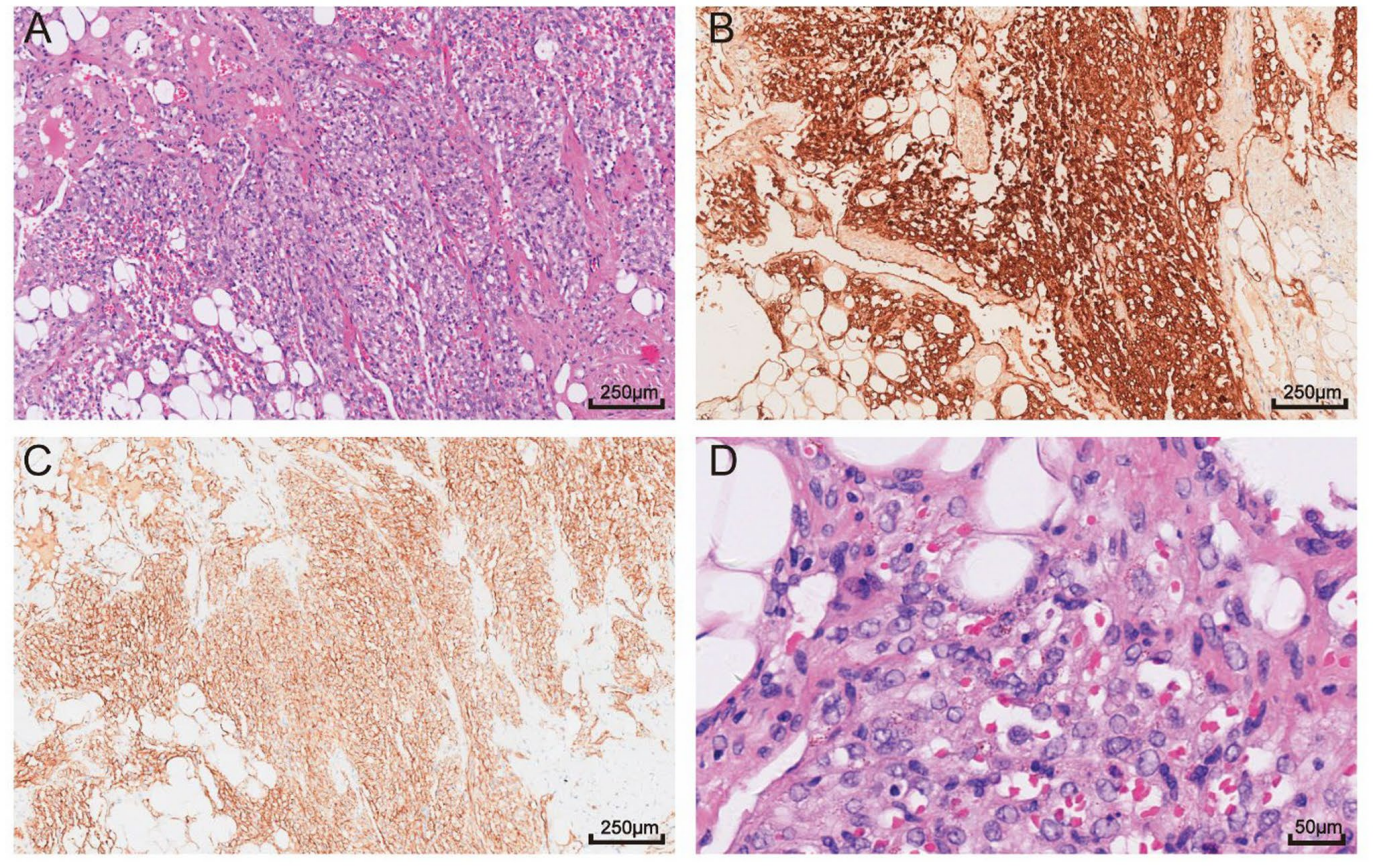
Histopathological features of a sigmoid mesenteric lesion in an adult patient with KLA. **(A)** Hematoxylin and eosin (H&E) staining (10×; scale bar = 250 μm). Irregular, sieve-like vascular structures are observed within the fibroadipose tissue of the sigmoid mesentery, without evident cytologic atypia. **(B)** D2-40 immunohistochemical staining (10×; scale bar = 250 μm). Endothelial cells within the lesion show positive expression, indicating lymphatic differentiation. **(C)** CD31 immunohistochemical staining (10×; scale bar = 250 μm). Both vascular and lymphatic endothelial cells within the lesion express CD31. **(D)** H&E staining (40×; scale bar = 50 μm). Focal aggregates of spindle-shaped cells are present, with fine brown-yellow granular deposits in the cytoplasm, consistent with hemosiderin accumulation associated with chronic hemorrhage.

Multiple percutaneous biopsies revealed abnormal vascular-like structures and lymphoid tissue proliferation with scattered spindle-shaped cells. Immunohistochemistry was positive for CD31, D2-40, and vimentin, confirming a lymphatic origin. Next-generation sequencing identified a somatic *NRAS* p.Q61R mutation with a variant allele frequency of 11.8%. Based on clinical, imaging, histopathological, and molecular findings, a definitive diagnosis of KLA complicated by DIC was established (Figure 1).

Sirolimus therapy was initiated at 1 mg/day in September 2016. Within three months, the patient’s diarrhea improved, imaging showed reduction in lesion size, and hematologic parameters began normalizing. Trough sirolimus levels were maintained at 4–5 ng/mL, and the drug was well tolerated, with only mild oral ulcers reported. A temporary dose escalation to 2 mg/day caused worsened oral ulcers without clinical benefit and was reduced back to 1 mg/day.

**Figure 2:**
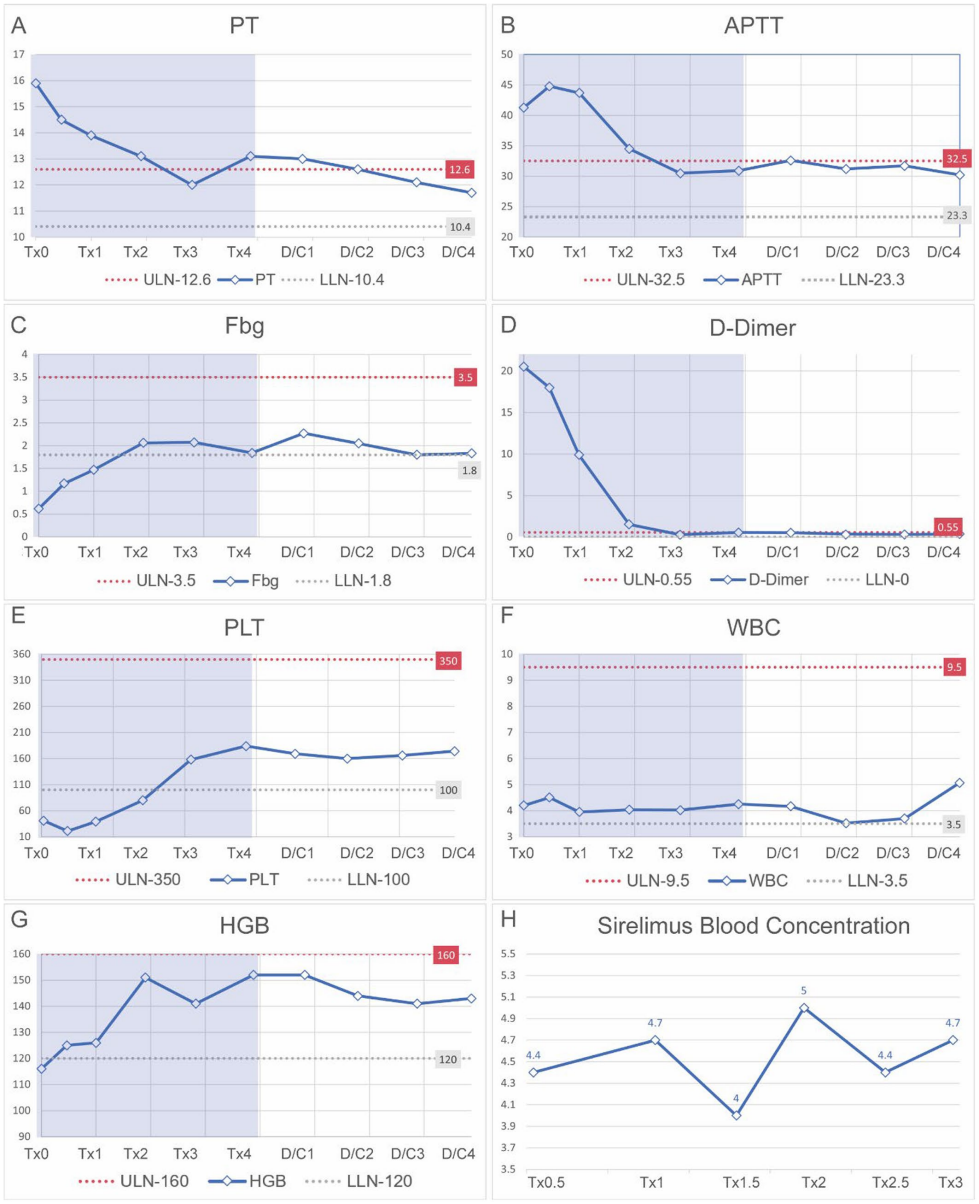
Coagulation, hematologic parameters, and sirolimus levels during and after treatment in a patient with KLA. The coagulation and hematologic abnormalities improved markedly after initiation of sirolimus therapy and remained stable following drug discontinuation, suggesting sustained long-term efficacy of low-dose sirolimus. **(A–G)** Coagulation and hematologic parameters. Tx indicates timepoints during sirolimus treatment; D/C indicates timepoints after drug discontinuation; ULN, upper limit of normal; LLN, lower limit of normal. X-axis unit: years. **(A)** Prothrombin time (PT, s). **(B)** Activated partial thromboplastin time (APTT, s). **(C)** Fibrinogen (Fbg, g/L). **(D)** D-dimer (mg/L FEU). **(E)** Platelet count (PLT, ×10^9^/L). **(F)** White blood cell count (WBC, ×10^9^/L). **(G)** Hemoglobin (HGB, g/L). **(H)** Sirolimus blood concentration (ng/mL).

**Figure 3:**
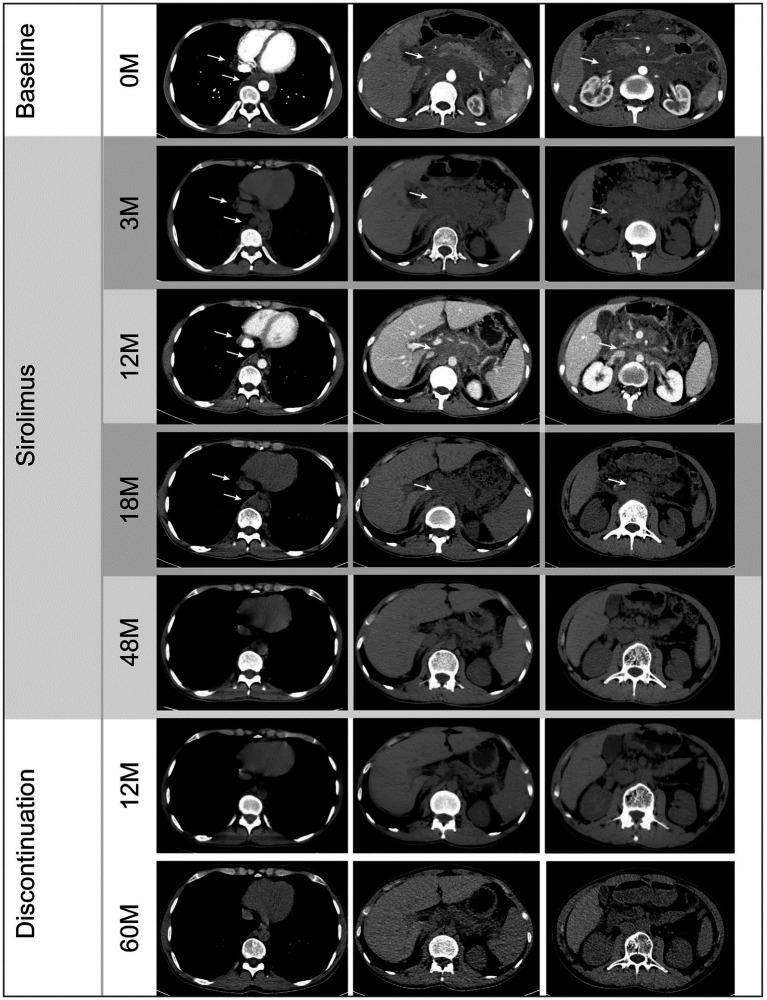
Longitudinal radiologic changes during treatment and follow-up in a patient with kaposiform lymphangiomatosis (KLA). Imaging timepoints include baseline (prior to treatment), sirolimus treatment (Tx) at 3, 12, 18, and 48 months, and post-treatment follow-up (D/C) at 12 and 60 months. “M” denotes months. Baseline CT revealed diffuse soft tissue infiltration in the mediastinum, retroperitoneum, and para-aortic regions. Arrows indicate representative lesions at each timepoint. Lesions gradually regressed during sirolimus treatment (3–48 M) and remained stable after discontinuation (12–60 M), indicating sustained disease control.

The patient continued long-term low-dose sirolimus for four years. Laboratory markers of DIC and blood counts normalized (Figure 2), and radiologic imaging demonstrated ongoing lesion regression (Figure 3). During treatment, only mild oral ulcers and mild acne were observed. Liver and renal function remained normal, and lipid profiles, including total cholesterol, triglycerides, high-density lipoprotein cholesterol, and low-density lipoprotein cholesterol, remained within the normal range. No severe infection, hyperlipidemia, hepatic or renal toxicity, or other treatment-limiting adverse events occurred. Sirolimus was discontinued in 2020, and the patient has remained relapse-free during five years of follow-up, highlighting sustained disease control and the potential feasibility of treatment withdrawal. A summary of the patient’s clinical course is presented in Table 1.

**Table 1:** Clinical timeline of the patient with kaposiform lymphangiomatosis.

Year	Clinical events
2013	Onset of chronic diarrhea.
2015	Colonoscopy performed; findings suggested nonspecific colitis. Treatment with mesalazine initiated but symptoms persisted.
2016	Progressive weight loss (~10 kg) and recurrent ecchymoses. Imaging revealed diffuse mediastinal and abdominal soft tissue lesions. Histopathology and genetic testing confirmed kaposiform lymphangiomatosis with NRAS p.Q61R mutation. Disseminated intravascular coagulation (DIC) was diagnosed.
September 16, 2016	Sirolimus initiated (1 mg/day).
2017	Clinical symptoms improved, laboratory abnormalities gradually normalized. Sirolimus dose temporarily increased to 2 mg/day.
2018	Dose reduced to 1 mg/day due to oral ulcers; disease remained stable.
2019–2020	Continued low-dose sirolimus therapy with stable trough levels (4–5 ng/mL) and sustained clinical remission.
2020	Sirolimus discontinued after approximately four years of treatment.
2020–2025	Regular follow-up showed stable imaging findings and normal coagulation parameters with no recurrence.

## Discussion

To our knowledge, this is one of the few reported adult cases of KLA demonstrating durable remission after long-term low-dose sirolimus therapy followed by successful drug discontinuation.

KLA is a rare and aggressive lymphatic anomaly characterized by diffuse lymphatic proliferation and a high incidence of disseminated intravascular coagulation (DIC). The disease is frequently associated with severe complications and poor prognosis (1, 16). Here, we describe an adult patient with molecularly confirmed KLA harboring an *NRAS* p.Q61R mutation, who achieved sustained disease control with long-term low-dose sirolimus and remained relapse-free for five years after drug discontinuation. Durable remission following sirolimus withdrawal has rarely been reported in KLA, particularly in adult patients (6, 14, 17–19).

Diagnosis of KLA remains challenging due to its heterogeneous clinical presentation and rarity. Clinical manifestations vary depending on the organs involved, and imaging findings frequently overlap with other lymphatic anomalies, including generalized lymphatic anomaly (GLA) and Gorham–Stout disease (2, 8, 20). Small biopsy samples may not always demonstrate the characteristic spindle-shaped lymphatic endothelial cells, leading to diagnostic uncertainty. In this case, multiple biopsies were performed before a definitive diagnosis was achieved. Histopathology revealed abnormal vascular-like structures with spindle cells, and immunohistochemistry was positive for lymphatic markers D2-40 and CD31, supporting a lymphatic origin. Importantly, next-generation sequencing identified the *NRAS* p.Q61R mutation, a known hallmark of KLA (7, 9, 16). The integration of clinical, pathological, and molecular evidence was essential for the final diagnosis.

Treatment of KLA remains challenging, as standardized guidelines are lacking. Conventional therapies, including corticosteroids, vincristine, interferon-*α*, and thalidomide, show limited and inconsistent efficacy (1, 21, 22). Surgical interventions may provide temporary symptom relief in selected cases but rarely alter disease progression and may worsen outcomes when extensive lesions are present (7). In the present case, operations are not recommended because of huge tumors and the available option of sirolimus, especially given the diffuse thoracoabdominal involvement, active DIC, and the potential risks of bleeding, lymphatic leakage, and procedure-related complications. In recent years, targeted therapies have advanced the management of complex lymphatic anomalies, with mTOR inhibitor sirolimus emerging as one of the most widely used systemic treatments (5, 13, 14). Retrospective studies and case series indicate that sirolimus can improve thrombocytopenia, correct coagulopathy, and reduce lymphatic effusions in many patients (14, 23–25). For example, a retrospective cohort of 24 patients with KLA reported that 58.3% achieved partial remission, and 25% maintained stable disease with sirolimus therapy, alongside significant improvement in platelet and fibrinogen levels (14).

Despite growing experience, several questions remain unresolved, including the optimal dose, therapeutic drug monitoring targets, duration of therapy, and feasibility of discontinuation. Most studies recommend trough concentrations of 10–15 ng/mL (1, 16, 26, 27), whereas others report effective ranges of 5–15 ng/mL, reflecting variability in practice (14). Sirolimus monotherapy may be insufficient in severe cases, necessitating combination therapy with corticosteroids, vincristine, propranolol, or bisphosphonates to control thrombocytopenia, bleeding, or bone involvement (1, 21, 22). Consequently, long-term therapy is often required, raising concerns about chronic immunosuppression and potential adverse effects.

In this case, significant clinical improvement was achieved with a relatively low dose of sirolimus (1 mg/day), yielding trough concentrations of 4–5 ng/mL, lower than typically recommended dosage (1, 14, 16, 24, 25). Temporary escalation to 2 mg/day increased adverse effects, particularly oral ulcers, without additional benefit. After returning to 1 mg/day, the patient maintained stable disease with normalization of hematologic and coagulation parameters. This suggests that effective disease control may be achievable at lower sirolimus exposure, potentially minimizing toxicity in selected patients.

Notably, after four years of continuous therapy, sirolimus was discontinued, and the patient has remained relapse-free for five years. Such long-term remission post-discontinuation is rarely described in KLA, where recurrence or drug dependence is commonly reported (6, 14, 19, 25). Compared with published cases requiring indefinite therapy or escalation, this observation supports careful treatment de-escalation in selected patients after prolonged stabilization.

To further clarify the basis for sirolimus discontinuation in this patient, the decision was made only after sustained disease control had been achieved following long-term therapy, rather than according to a standardized withdrawal protocol, as no established guideline currently defines the optimal timing of sirolimus discontinuation in KLA. Before discontinuation, the lesions had markedly decreased in size, the patient remained clinically stable without recurrent symptoms, DIC-related abnormalities had returned to the normal range, and complete blood counts remained stable. These findings suggested that the disease had entered a stable and well-controlled state. Therefore, after an individualized risk–benefit assessment, particularly considering the young age of the patient and the potential burden of prolonged treatment, sirolimus discontinuation was attempted. Discontinuation may be considered only in selected patients who have achieved sustained clinical, laboratory, and radiological stability after long-term treatment.

Given the uncertainty of the long-term disease course after sirolimus withdrawal in KLA, regular follow-up is essential after discontinuation. Follow-up should focus on early detection of disease recurrence or progression through comprehensive clinical, laboratory, and radiological assessment. Clinical symptoms and signs should be monitored carefully, together with complete blood counts and coagulation parameters, including PT, APTT, fibrinogen, and D-dimer. Serial chest and abdominal imaging should also be continued to evaluate changes in lesion size and disease extent. If symptoms recur, coagulation abnormalities reappear, or imaging suggests disease progression, re-initiation of sirolimus or other targeted therapy should be reconsidered.

Radiological follow-up is particularly important in this setting, as imaging provides direct evidence of lesion stability or progression after drug discontinuation. MRI has important advantages in the assessment of soft tissue lesions and can reduce radiation exposure, especially in pediatric patients requiring long-term surveillance. CT was selected for follow-up in this case because of its rapid accessibility in clinic and practical value in evaluating the size and extent of thoracoabdominal lesions.

Therapeutic responses to sirolimus are heterogeneous. Some *NRAS*-mutated KLA patients show partial response or progression despite treatment (3, 6, 19, 23). As *NRAS* mutations activate both the PI3K/AKT/mTOR and RAS/MAPK pathways, mTOR inhibition alone may not fully suppress disease (11, 12). MEK inhibitors, such as trametinib, have emerged as promising alternatives, improving thrombocytopenia and pulmonary function in *NRAS*-mutant KLA (9). Experimental studies indicate that MEK inhibition can suppress spindle-shaped morphological changes in lymphatic endothelial cells induced by *NRAS* mutations (12, 28, 29).

Beyond *NRAS* p. Q61R, other mutations affecting RAS–MAPK and PI3K pathways, including *CBL*, *HRAS*, and *PIK3CA*, have been reported in complex lymphatic anomalies (30). These findings highlight molecular heterogeneity and underscore the importance of genetic testing for targeted and personalized therapy.

The main limitation of this report is that it describes a single case. Nevertheless, our center has extensive experience using sirolimus in lymphangioleiomyomatosis (LAM), where relatively low sirolimus concentrations yield comparable benefits with fewer adverse effects (31–33) The favorable long-term outcome observed here supports the potential value of individualized low-dose sirolimus therapy in selected lymphatic disorders.

## Conclusion

This case demonstrates that long-term low-dose sirolimus therapy effectively controlled disease progression in adult kaposiform lymphangiomatosis complicated by disseminated intravascular coagulation. In this patient, sirolimus was administered at a low dose with trough concentrations of 4–5 ng/mL for approximately four years. After sustained clinical, laboratory, and radiologic remission, sirolimus was discontinued, and the patient remained relapse-free during long-term follow-up. Careful monitoring may allow treatment discontinuation with sustained remission in selected patients.

### Clinical message

Treatment with low-dose sirolimus can achieve sustained disease control in patients with kaposiform lymphangiomatosis, and treatment discontinuation may be feasible in well-controlled patients.

### Patient perspective

Patients with kaposiform lymphangiomatosis may benefit from long-term low-dose treatment of sirolimus, and discontinuation of sirolimus is feasible in appropriately selected patients.

## Data Availability

The data supporting the conclusions of this article will be made available by the corresponding author upon reasonable request.
